# Pediatric eosinophilic granulomatosis with polyangiitis and intracardiac thrombus: A case report

**DOI:** 10.1177/2050313X241309966

**Published:** 2024-12-19

**Authors:** Katharine V. Jensen, Nicholas Brochez, Christopher Spence, Joel Livingston, Michael Khoury, Jeanine McColl

**Affiliations:** 1 Department of Pediatrics, Faculty of Medicine & Dentistry, University of Alberta, Edmonton, AB, Canada; 2 Division of General Pediatrics, Stollery Children’s Hospital, Edmonton, AB, Canada; 3 Division of Cardiology, Stollery Children’s Hospital, Edmonton, AB, Canada; 4 Division of Haematology/Oncology, Stollery Children’s Hospital, Edmonton, AB, Canada; 5 Division of Rheumatology, Stollery Children’s Hospital, Edmonton, AB, Canada

**Keywords:** EGPA, pediatrics, refractory asthma, vasculitis, intracardiac mass

## Abstract

Eosinophilic granulomatosis with polyangiitis (EGPA) is a rare systemic necrotizing vasculitis marked by eosinophilia and extravascular granulomas, predominantly affecting the respiratory tract. This report details a unique EGPA case in a 6-year-old girl with extensive cardiac involvement, featuring an atypical intracardiac mass suggestive of endomyocardial fibrosis and a concomitant thrombus. The clinical course unfolded in three phases: an initial prodrome with asthma; subsequent peripheral hypereosinophilia; and ultimately systemic vasculitis. Cardiac involvement, notably an intracardiac mass in the right ventricular apex extending into the interventricular septum, underscored the diverse nature of EGPA. The patient fulfilled sufficient criteria outlined by the American College of Rheumatology and the European Alliance of Associations for Rheumatology for an EGPA diagnosis, displaying hypereosinophilia, obstructive airway disease, and biopsy-confirmed inflammation predominantly characterized by extravascular eosinophils. Treatment included high-dose methylprednisolone and cyclophosphamide, which resulted in clinical improvement and inflammatory marker normalization. To halt right ventricular thrombus progression, therapeutic unfractionated heparin was initiated, and she was transitioned to warfarin, which resulted in complete resolution of the cardiac mass. This case highlights the necessity of a multidisciplinary approach for managing complex EGPA manifestations, particularly in pediatrics, and emphasizes the importance of timely intervention in mitigating the impact of cardiac complications associated with EGPA.

## Introduction

Eosinophilic granulomatosis with polyangiitis (EGPA) is a rare and potentially life-threatening vasculitis with a reported prevalence of 10–13 per million in adults,^1^ with even lower pediatric prevalence rates.^2–4^ EGPA is characterized by eosinophil-rich, necrotizing granulomatous inflammation within the small to medium vessels in the respiratory tract and other organ systems.^5,6^ The most common extrapulmonary manifestations include cutaneous, cardiac, and gastrointestinal.^1,4,6–8^ EGPA is typically seen in adults with a mean age of disease onset of 50 years.^3,7^ In pediatric patients, disease onset is typically in the second decade,^4^ and tends to be associated with higher morbidity and mortality.^2–4,9^ Median time to diagnosis from symptom onset is 2 years.^9^

EGPA characteristically presents in three phases.^1,4,7,10^ First, there is typically a prodrome of asthma, allergic rhinitis, and/or nasal polyps. Respiratory symptoms may begin years before other symptoms develop. Next, there is development of peripheral hypereosinophilia with extravascular eosinophils and pulmonary infiltrates. The third phase is systemic vasculitis, which is often preceded by symptoms such as fever, myalgias, and arthritis.^1,2,4,7^ Any organ system can be affected in this phase, but cutaneous, cardiac, and gastrointestinal involvements are most common.

The American College of Rheumatology (ACR)/European Alliance of Associations for Rheumatology (EULAR) EGPA classification criteria should be considered in patients with small or medium vessel vasculitis and require a cumulative score of ⩾6 points based on the following criteria: obstructive airway disease (+3 points), nasal polyps (+3 points), mononeuritis multiplex (+1 point), eosinophilia ⩾1 × 10^9^/L (+5 points), extravascular eosinophilic-predominant inflammation on biopsy (+2 points), cytoplasmic antineutrophil cytoplasmic antibodies (cANCAs) or anti-proteinase 3 (anti-PR3) antibody positivity (−3 points), and hematuria (−1 point).^5^

Despite its rarity in children, understanding EGPA’s clinical presentation and disease impact is vital, given its challenging diagnostic nature and the absence of robust evidence regarding treatment in the pediatric population. It is a challenging diagnosis to make, requiring a high index of suspicion. High-quality evidence regarding treatment of EGPA in children is lacking. In this context, we present a case of a 6-year-old girl with newly diagnosed EGPA and extensive cardiac involvement. As the pediatric population faces heightened morbidity and mortality risks, increased awareness of EGPA is crucial for prompt diagnosis and intervention.

## Case

A 6-year-old female presented to the pediatric emergency department with a 2-month history of transient arthralgias, fever, abdominal pain, painful cutaneous nodules, and weight loss. She then developed a petechial rash 1 month prior to presentation that started on the face and progressed to the entire body, including the oral mucosa. Her past medical history was significant for refractory asthma that had been diagnosed after a symptomatic COVID-19 infection 1 year prior. She was initiated on beclomethasone 100 mcg two puffs twice daily and salbutamol as needed. In 1 year, she had 18 presentations to the pediatric emergency department with respiratory symptoms and four admissions to hospital. After her second hospitalization for an asthma exacerbation, a leukotriene receptor antagonist, montelukast 5 mg oral daily, was added. Subsequently, treatment was escalated to medium dose inhaled corticosteroid/long-acting beta agonist with 100 mcg mometasone furoate/5 mcg formoterol fumarate dihydrate two puffs twice daily following her third admission for an asthma exacerbation. With each hospitalization, she received 5 days of 2 mg/kg prednisone. Ten days prior to the presentation that led to her EGPA diagnosis, she was started on amoxicillin–clavulanic acid for an episode of hemoptysis in the context of refractory respiratory symptoms following oral steroid treatment for an asthma exacerbation. Pulmonary function tests revealed severe obstructive airway disease with bronchodilator responsiveness (FEV1 49% predicted with 22% bronchodilator response, forced vital capacity (FVC) 66%, FEV1/FVC 0.68, total lung capacity (TLC) 79%).

On the presentation that led to an EGPA diagnosis, bloodwork demonstrated normocytic anemia (Hgb 108 g/L), leukocytosis (white blood cell (WBC) 24.9 × 10^9^/L), neutrophilia (10.5 × 10^9^/L), peripheral eosinophilia (8.9 × 10^9^/L), and elevated C-reactive protein (46.7 mg/L). Chest X-ray showed small airway inflammation in keeping with asthma. ANCA, anti-nuclear antibody (ANA), anti-extractable nuclear antigen (anti-ENA), anti-MPO (anti-myeloperoxidase), anti-dsDNA (anti-double-stranded DNA), and antiphospholipid antibodies were negative. An abdominal ultrasound was performed due to complaint of severe abdominal pain and intermittent intussusception was observed.

**Figure 1.**
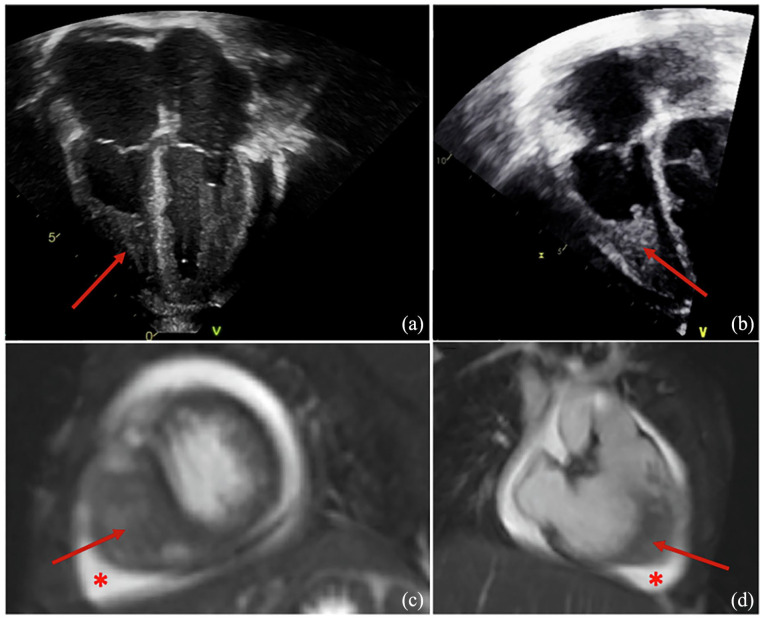
(a and b) Echocardiogram apical images showing RV mass occupying much of the RV apex. (c and d) Cardiac MRI showing RV mass and pericardial effusion. Arrow: RV mass; asterisk: pericardial effusion; RV: right ventricle; MRI: magnetic resonance imaging.

**Figure 2.**
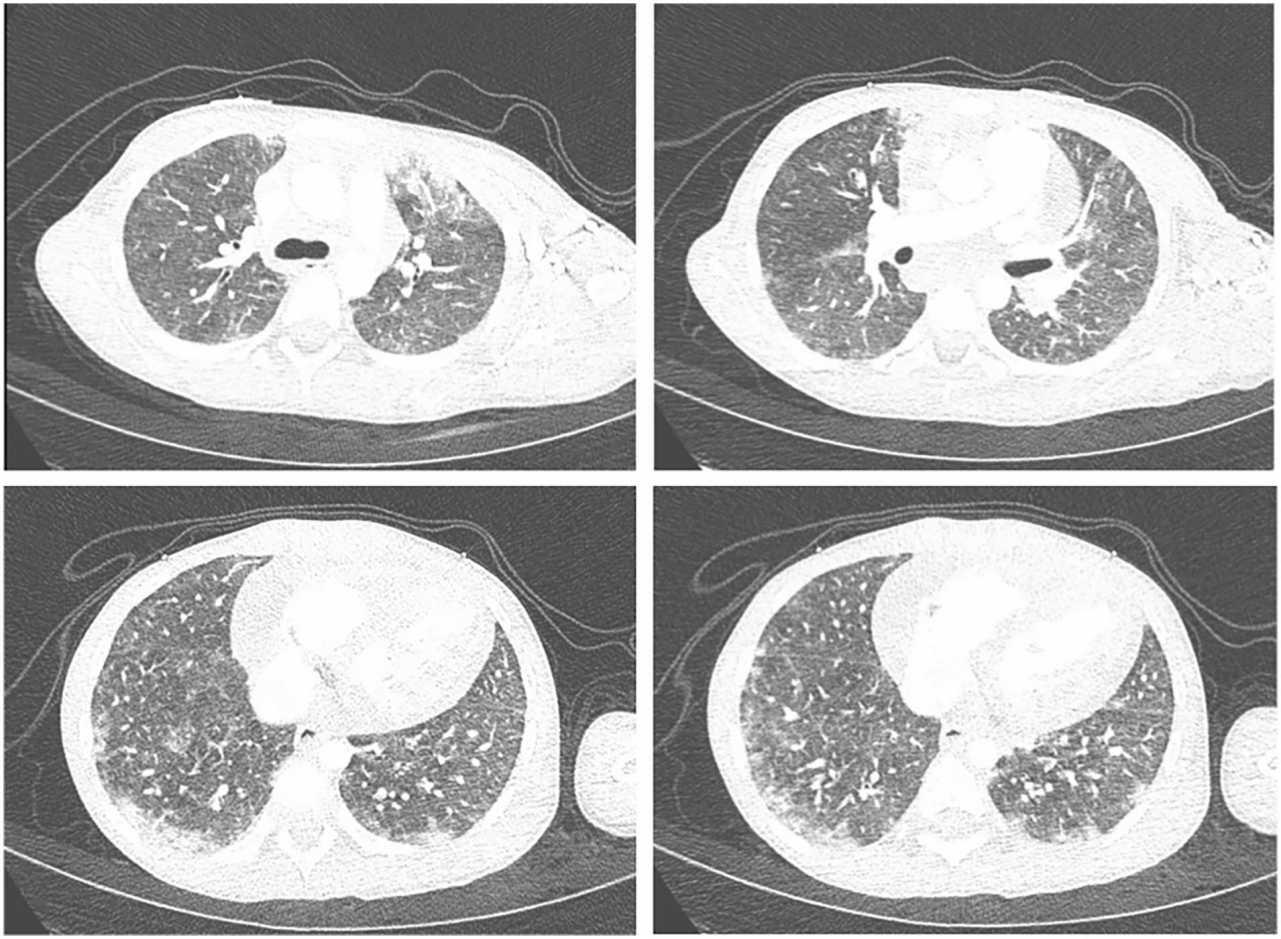
Diffuse patchy ground glass opacification in a predominantly subpleural distribution, most striking at both the posterior aspect of the lung bases and the anterior aspect of the lung apices.

On admission, she had unexplained persistent tachycardia. High-sensitivity troponin was 557 ng/L (normal < 14 ng/L). An echocardiogram showed a moderate pericardial effusion and the right ventricle (RV) cavity appeared foreshorten by echogenic material filling much of the apex (Figure 1(a) and (b)). It was not initially clear whether this was dense trabeculation, thrombosis, or other echogenic material. A CT angiogram revealed bilateral pulmonary ground glass opacities peripherally (Figure 2), mildly dilated pulmonary arteries, and an RV intracavitary lesion suspected to be thrombus. A therapeutic unfractionated heparin (UFH) infusion was started with daily monitoring of anti-Xa level (target 0.34–0.7). Cardiac magnetic resonance imaging (MRI) indicated that the intracavitary contents within the RV were suggestive of endomyocardial fibrosis, with extension into the interventricular septum, with pericardial effusion (Figure 1(c) and (d)) and signs suggestive of myopericarditis. Overall, given her hypereosinophilia it was felt that the RV mass likely had components of endomyocardial fibrosis which were a nidus for secondary thrombus.

During hospitalization, her peripheral eosinophil count peaked at 18.1 × 10^9^/L. A bone marrow aspirate and biopsy were performed before starting on steroids and were negative for neoplasia yet they revealed marked eosinophilia (39%). Karyotype and molecular testing on the marrow, including platelet-derived growth factor receptor alpha (PDGFRA), were unremarkable. A skin biopsy of the lower back was consistent with small vessel vasculitis with prominent eosinophilia.

According to ACR/EULAR criteria,^5^ she met classification for EGPA with a score of 9. She received points for hypereosinophilia (+5), obstructive airway disease (3+), and extravascular eosinophilic-predominant inflammation on biopsy (+2). Points were deducted for hematuria (−1).^5^ Induction treatment included high dose of IV methylprednisolone with stepdown to oral prednisone. Given the cardiac involvement, she was treated as per the ACR guidelines for remission induction for active, severe disease with intermittent cyclophosphamide; she received 500 mg/m^2^ every 2 weeks for three doses, followed by 500 mg/m^2^ every 3 weeks for three doses.^11^ Cyclophosphamide was administered on day 7 of steroid treatment. With initiation of pulsed steroids, there was clinical improvement and normalization of eosinophilia and inflammatory markers. She was transitioned from UFH to warfarin (international normalized ratio (INR) target 2–3). In follow-up, less than 3 months later, echocardiogram revealed that the RV mass—including components of thrombus—had completely resolved.

Written informed consent was obtained from the caregiver of the patient prior to documenting this case report.

## Discussion

We describe a unique case of EGPA in a 6-year-old female with extensive cardiac involvement. To the best of our knowledge, this is the first report of a pediatric patient with EGPA with a mass in the RV suggestive of endomyocardial fibrosis and thrombus. Symptomatic cardiac involvement occurs in 27%–47% of EGPA^9^ and cardiac complications are often responsible for early death and poor long-term prognosis, with childhood mortality rates as high as 15%.^9^

As was the case in this patient, cardiac involvement is more prevalent in those who are ANCA negative and have higher eosinophilia.^12,13^ Cardiac manifestations in EGPA vary in severity and may include pericardial effusion, valve insufficiency, ventricular dysfunction, cardiomyopathy, pulmonary hypertension, and thrombosis.^12^ Cardiac MRI may further delineate endomyocarditis, secondary cardiomyopathy, and thrombus.^12,14^ Electrical disturbances may also arise, including bundle branch blocks, ST and T wave abnormalities, and ventricular arrhythmias.^12^ Early detection of cardiac involvement is crucial as it carries a poor prognosis.^7,15^ As such, early and periodic screening with electro- and echocardiography, high-sensitivity troponin I, and N-terminal pro-brain natriuretic peptide is recommended. Cardiac MRI should be considered early in those with overt cardiac manifestations on screening echocardiogram.^13,16^ Early treatment of hypereosinophilia with steroids and anticoagulation therapy to prevent thrombus progression is imperative given cardiac involvement in childhood EGPA carries a pessimistic prognosis.

This case highlights that it is essential to consider EGPA in the differential for refractory asthma, as EGPA can initially present with a prodromal phase of difficult-to-control asthma. Our patient had 18 presentations to care for uncontrolled asthma before a diagnosis was made. Mepolizumab, an interleukin-5 (IL-5) inhibitor, used to reduce eosinophil levels in severe eosinophilic asthma, may provide additional benefit in controlling other manifestations of hypereosinophilia as an adjunct therapy during the maintenance phase after completing cyclophosphamide induction. However, it is not on-label for EGPA alone in pediatrics. A previous study demonstrated successful treatment of relapsing EGPA with mepolizumab in a pediatric patient.^17^ Mepolizumab has been studied and approved for on-label use in adults with EGPA and has contributed to reduced relapse rates and reduced need for systemic steroids.

In addition, the recent FDA approval of benralizumab for adult patients with EGPA^18^ presents a promising new management option. Benralizumab, which also targets IL-5 signaling to reduce eosinophil levels, may offer complementary benefits, particularly for patients with refractory symptoms and those who have not achieved adequate control with other therapies. Its role in pediatric populations remains to be established, but ongoing research may illuminate its potential utility in this demographic as well.

More common small vessel vasculitides were considered in the differential, including IgA leukocytoclastic vasculitis, given the abdominal pain, arthralgias, petechial rash, hematuria, and intussusception. It did not explain the long-standing difficulty to treat asthma or peripheral eosinophilia, and the skin biopsy was not supportive.

## Conclusion

EGPA poses a rare and potentially life-threatening challenge, particularly in pediatric cases, where the morbidity and mortality rates surpass those observed in adults. The current understanding of EGPA in children is hindered by limited data, leading to the adoption of treatment strategies extrapolated from adult cases, thereby lacking standardization in pediatric care. The pressing need for refining early identification and comprehension of pediatric EGPA is evident.
